# Mediator complex (MED) 7: a biomarker associated with good prognosis in invasive breast cancer, especially ER+ luminal subtypes

**DOI:** 10.1038/s41416-018-0041-x

**Published:** 2018-03-28

**Authors:** Chitra Joseph, Olivia Macnamara, Madeleine Craze, Roslin Russell, Elena Provenzano, Christopher C. Nolan, Maria Diez-Rodriguez, Sultan N. Sonbul, Mohammed A. Aleskandarany, Andrew R. Green, Emad A. Rakha, Ian O. Ellis, Abhik Mukherjee

**Affiliations:** 1 Division of Cancer and Stem Cells, School of Medicine, University of Nottingham and Nottingham University Hospitals NHS Trust, City Hospital Campus, Nottingham, NG5 1PB UK; 2 CRUK Cambridge Research Institute, Cambridge, UK; 3 Addenbrooke’s Hospital, Cambridge University Hospital NHS Foundation Trust, Cambridge, UK

**Keywords:** Breast cancer, Biomarkers

## Abstract

**Background:**

Mediator complex (MED) proteins have a key role in transcriptional regulation, some interacting with the oestrogen receptor (ER). Interrogation of the METABRIC cohort suggested that MED7 may regulate lymphovascular invasion (LVI). Thus MED7 expression was assessed in large breast cancer (BC) cohorts to determine clinicopathological significance.

**Methods:**

MED7 gene expression was investigated in the METABRIC cohort (*n* = 1980) and externally validated using bc-GenExMiner v4.0. Immunohistochemical expression was assessed in the Nottingham primary BC series (*n* = 1280). Associations with clinicopathological variables and patient outcome were evaluated.

**Results:**

High MED7 mRNA and protein expression was associated with good prognostic factors: low grade, smaller tumour size, good NPI, positive hormone receptor status (*p* < 0.001), and negative LVI (*p* = 0.04) status. Higher MED7 protein expression was associated with improved BC-specific survival within the whole cohort and ER+/luminal subgroup. Pooled MED7 gene expression data in the external validation cohort confirmed association with better survival, corroborating with the protein expression. On multivariate analysis, MED7 protein was independently predictive of longer BC-specific survival in the whole cohort and Luminal A subtype (*p* < 0.001).

**Conclusions:**

MED7 is an important prognostic marker in BC, particularly in ER+luminal subtypes, associated with improved survival and warrants future functional analysis.

## Introduction

Breast cancer (BC) is a heterogeneous disease varying in presentation, morphological types, response to therapy and patient outcome. The development of high-throughput technologies to investigate genetic, epigenetic and proteomic changes has helped to unravel the complexity of BC biology.^1^ Metastasis is the major cause of morbidity and mortality in BC patients, and lymphovascular invasion (LVI) rather than blood vascular invasion seems to be the major mechanism involved in the early stages of metastases in BC.^2^ LVI has been shown to correlate with an increased histological grade and a poor prognosis.^3^ The molecular mechanisms of LVI are complex, involving multiple pathways, and remain hugely unknown.^4^ The Molecular Taxonomy of Breast Cancer International Consortium (METABRIC) study investigated the genomic and transcriptomic data across 2000 breast tumours, where associations between germline variants (copy number variations and single-nucleotide polymorphisms), somatic aberrations (copy number aberrations (CNAs)) and alterations in gene expression were found. Further clustering analyses have identified 10 novel clusters (IntClusts 1–10), which were associated with distinct CNA and gene expression changes. The clusters have further divided the molecular subtypes and were associated with different clinical outcomes and drivers. In this study, a novel approach was utilised to unravel the molecular determinants of LVI. Global gene expression profiling data from the METABRIC cohort^5^ was utilised to identify differentially expressed genes related to LVI status as determined by central histopathology review of haematoxylin and eosin (H&E) sections supplemented by D2-40 immunohistochemical (IHC) analysis. Among the top differentially expressed genes, the Mediator protein MED7 was found to be inversely associated with LVI.

The Mediator complex is a multi-subunit transcriptional coactivator complex required for the transcription of nearly all protein-coding genes.^6^ By its direct association with both signal-activated transcription factors and the RNA polymerase II transcription machinery, the Mediator proteins function as general integrators of regulatory signals that congregate on the promoters of protein-coding genes.^7^ Disruption to Mediator subunits can affect many different cellular functions and fates, some of which could potentially be involved in carcinogenesis.^8^ One of the Mediator subunits; MED7, plays an important role in gonadal development and embryogenesis.^9^ However, despite being a highly conserved Mediator subunit, MED7 is not essential for viability across all species. A study investigating the functions of MED7 in *Candida albicans* found that a loss of MED7 did not lead to loss of viability.^10^ However, loss of MED7 has been reported to have a substantial impact on several cellular functions, in particular, impairing metabolic functions. Overall, by deletion of different Mediator sub-modules, including MED7N (the N-terminal subunit of MED7), metabolic sensing, stress response and certain amino-acid biosynthesis pathways are affected.^11^ Gene expression profiling in gastrointestinal stromal tumours has shown that MED7 downregulation is associated with an increased tumour risk and could therefore potentially be a marker of favourable prognosis.^12^ Other members of the Mediator complex such as MED1 have been reported to be associated with LVI in other series^13^ and hence MED7, another member of the Mediator complex, was deemed to be an interesting candidate to investigate. MED1 and MED24 have also been found to interact to mediate oestrogen receptor (ER) functions and regulate pubertal mammary gland and BC development.^14^ Because of the association of other Mediator proteins with ER, the role of MED7 in ER+ BCs was also explored, as ER+ BCs remain the most heterogeneous molecular group.^15, 16^ Overall, this study aimed to investigate the clinicopathological and biological significance of MED7 in BC including its role in LVI and hormonal receptor status.

## Materials and methods

### Differential gene expression and the selection of MED7

LVI status, as defined by morphology (H&E) supplemented with IHC (D2-40 and CD34), was available for the Nottingham subset of the METABRIC cohort and hence interrogated for differences between LVI+ and LVI− subgroups. Lymph node (LN)-positive samples were excluded from the LVI− subgroup to avoid the confounding effect of undetectable LVI in these tumours. Data on differentially expressed genes between LVI+ and LVI− subgroups was obtained from microarray analysis data normalised to fit into a linear regression model (LIMMA: linear models of microarray analysis). *MED7* was ranked within the top 10% of these candidate genes as inversely (log fold change: −1.29) correlated with LVI (adjusted *p*-value = 0.0005). Chosen as a gene of interest, data from the whole (METABRIC) cohort (Supplementary Table 1; *n* = 1980)^5^ was used to evaluate the mRNA expression of MED7. [The METABRIC study provides data on genomic and transcriptomic profiling of BC using the Affymetrix SNP 6.0 and Illumina HT-12 v3 platforms, respectively. Detailed description of the experimental assays and analytical methods used have been described previously].^5, 17^ The assessment of the clinicopathological impact of *MED7* transcription and its associations with clinical outcome in the whole METABRIC series was performed by setting a cut-off point for the mRNA expression of *MED7* at the median.

### External validation cohort

For external validation, *MED7* mRNA expression was interrogated through the BC-GenExMiner v4.0 (Breast Cancer Gene-Expression Miner v4.0 online data set (http://bcgenex.centregauducheau.fr)^18^, also used in other published studies.^19^ This is composed of two statistical mining modules: the ‘prognostic module’, offering the possibility to evaluate the *in vivo* prognostic impact of candidate genes in BC, and the ‘correlation module’, to compute correlation coefficients between gene expressions or to find lists of correlated genes in BC. In this external validation, the prognostic module of the BC-GenExMiner, which evaluates the *in vivo* prognostic impact of candidate genes in BC was utilised, using Cox model and Kaplan–Meier plot generation.^18^ Data sets with available MED7 expression in this online repository are catalogued in Supplementary Table 2.

### Patients and tumours

The well-characterised Nottingham Tenovus Primary BC Series comprised the study population for protein expression (Supplementary Table 3). Briefly, this comprised of women aged ≤70 years who presented to Nottingham City Hospital from 1988 to 1998 and received uniform treatment with a long-term follow-up period. Patients’ clinicopathological profiles included histological phenotype, molecular subtypes, primary tumour size, histological grade, tumour stage, nodal status, Nottingham Prognostic Index (NPI), receptor status and other BC-related biomarkers.^20, 21^ Outcome data including BC-specific survival (BCSS) and distant metastasis-free interval (MFI) was maintained on a prospective basis. BCSS was defined as the interval (in months) from the date of primary surgery to the time of death because of BC, while MFI identified as the interval from the date of primary surgery to the appearance of distant metastasis.

### Western blotting

For validation of MED7 rabbit monoclonal antibody [EPR15410 (Abcam, Ab187146, Cambridge, UK] specificity, western blotting was performed on whole-cell lysates of MCF-7, SKBr3 and HEK293 (obtained from the American Type Culture Collection; Rockville, MD, USA) cell lines using 1:1000 dilution of the primary antibody and fluorescent secondary antibodies (1:15,000) (IR Dye 800CW donkey anti-rabbit and 680RD donkey anti-mouse, LI-COR Biosciences, UK). Five percent milk (Marvel Original Dried Skimmed Milk, Premier Food Groups Ltd, St Albans, UK) was used for blocking. Mouse β-Actin (A5441, Sigma-Aldrich; Clone AC-15; Sigma, UK) at 1:5000 was used as a house-keeping protein. A protein ladder (Page Ruler Plus Prestained Protein Ladder, ThermoScientific, Waltham, MA, USA) was included. To visualise bands, fluorescence at wavelengths of 600, 700 and 800 nm was used on a Licor Odyssey Fc with image studio 4.0 (LI-COR Biosciences).

### Protein expression by IHC

Tumour samples were arrayed onto tissue microarrays (TMAs) as previously described.^20^ IHC was performed on TMA sections using the Novolink Max Polymer Detection system (Leica, Newcastle, UK). In brief, sections were deparaffinised with xylene and rehydrated through 100% ethanol. Heat-induced retrieval of antigen epitopes was performed in citrate solution (pH 6.0). MED7 staining was performed with a rabbit monoclonal antibody [EPR15410 (Abcam- Ab187146, Cambridge, UK], diluted (1:50) and incubated for 60 min at room temperature. 3-3’ Diaminobenzidine tetrahydrochloride (Novolink DAB substrate buffer plus) was freshly prepared and used as chromogen. Counter staining was performed using Meyer’s haematoxylin for 6 min. Negative (omission of the primary antibody) and positive controls (anti-human-β-2-microglobulin; A0072, Dako) were included.

The modified *H*-score method was used in assessing IHC staining, taking the staining intensity and percentage positivity into account.^22^ Briefly, the percentages of positively stained tumour cells for each of these intensities were subjectively estimated. Staining intensity (0–3) was multiplied by percentage (0–100) and final scores were obtained, giving a range of 0–300. High-resolution digital images were generated via scanning the IHC-stained slides (Nanozoomer; Hamamatsu Photonics, Welwyn Garden City, UK) at ×20 magnification to facilitate the scoring of the TMA cores using a high-resolution (1920 × 1080) screen. Staining was double scored blindly by two researchers including a consultant histopathologist for ~25% cores to assess interobserver concordance. IHC staining and dichotomisation of the other biomarkers included in this study were as per previous publications^21, 23–30^ (Supplementary Table 4). BC molecular subtypes were defined based on the IHC profile as: Luminal A: ER+/HER2− low proliferation (Ki67 < 10%), Luminal B: ER+/HER2− high proliferation (Ki67 ≥ 10%), HER2-positive class: HER2+ regardless of ER status, triple negative (TPN): ER−, PgR− and HER2−.

### Statistical analysis

IBM SPSS 22.0 (Chicago, IL, USA) software was used for statistical analysis. Univariate analysis was performed using the chi-squared test to evaluate the significance of the association between expression of the biomarkers and the clinicopathological parameters of the data, as well as other previously investigated biomarkers. Kaplan–Meier analysis with a log-rank test for significance was performed to assess BCSS and disease-free interval survival differences. Multivariate Cox Regression analysis with adjustment of covariates was fitted to test independence from standard prognostic factors in BC (stage, grade and LVI). A *p*-value of <0.05 was considered significant.

### Ethics

This study was approved by the Nottingham Research Ethics Committee 2 under the title ‘Development of a molecular genetic classification of breast cancer’. All samples from Nottingham used in this study were pseudo-anonymised and collected prior to 2006 and therefore under the Human Tissue Act informed patient consent was not needed. Release of data was also pseudo-anonymised as per Human Tissue Act regulations.

## Results

### MED7 mRNA expression and clinicopathological parameters

**Fig. 1:**
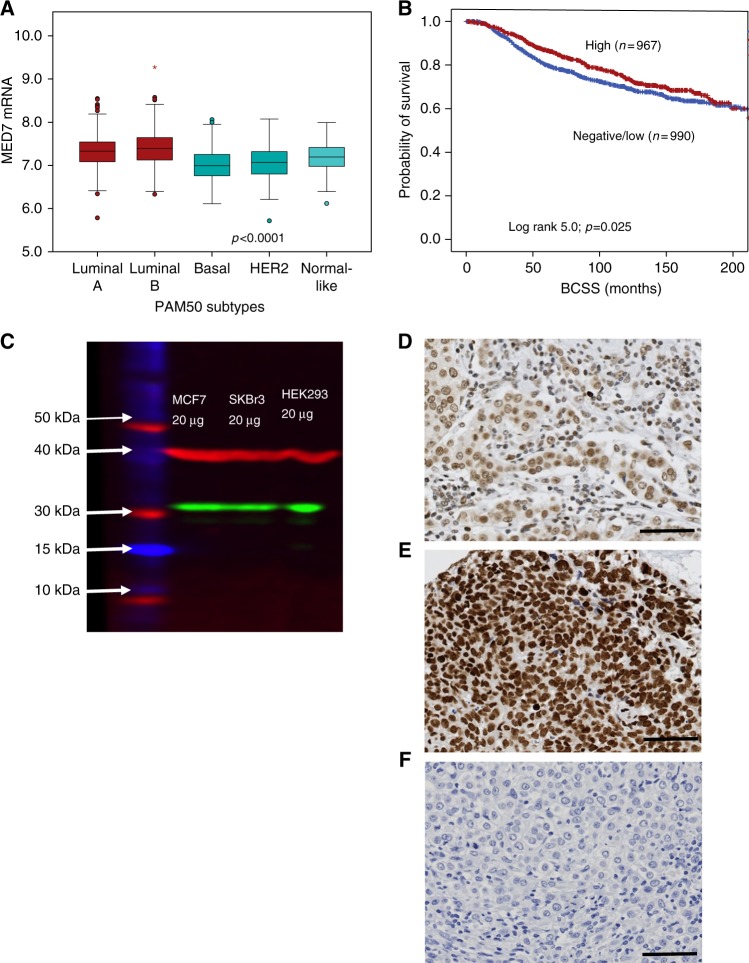
**a** MED7 mRNA and PAM50 subtypes, **b** MED7 mRNA vs BCSS in the whole cohort and **c** western blotting analysis using MED7 rabbit monoclonal antibody [EPR15410]. Intensity levels of staining are shown: **d** low, **e** strong, and **f** negative expression (×200 magnification)

In the METABRIC cohort, high *MED7* mRNA expression was observed in 981 cases (49.6%). High *MED7* expression was associated with lower grade (*p* < 0.0001), older age (*p* < 0.0001) and good/excellent prognostic histological types (lobular and mucinous types (*p* = 0.001). It was also associated with ER+ and progesterone receptor-positive (PR+) tumours (both *p* < 0.0001) and HER2-negative status (*p* = 0.00001; Table 1). When comparing the levels of *MED7* mRNA expression in the intrinsic (PAM50) subtypes, significant correlations were observed with luminal subtype tumours while the basal subtype showed the least expression levels (*p* < 0.0001; Fig. 1a). High *MED7* mRNA expression was significantly associated with IntClusts 3 and 8 (*p* < 0.00001), clusters known to be enriched for Luminal A-like BCs associated with the most favourable clinical outcome in the METABRIC cohort. Overexpression of *MED7* mRNA displayed significantly improved patients’ survival in the whole cohort (Fig. 1b; *p* = 0.025). There was no association between *MED7* mRNA expression and outcome in any of the PAM50 subtypes (Supplementary Figure 1A-D). As stated in the materials and methods, *MED7* was inversely (log fold change: −1.29) correlated with LVI (adjusted *p*-value = 0.0005) when interrogated on the Nottingham subset of the cohort, that had available LVI data.

**Table 1:** Associations between MED7 mRNA expression and clinicopathological variables in the METABRIC cohort

Clinicopathological criteria	MED7 mRNA expression		χ^2^ (*p*-value)
	Low (%)	High (%)	
Age at diagnosis (years)			
≤50	251 (59.3)	172 (40.7)	17.35 (<**0.00001**)
>50	723 (47.9)	787 (52.1)	
Tumour size (cm)			
≤2.0	423 (49.4)	434 (50.6)	0.36 (0.554)
>2.0	558 (50.7)	542 (49.3)	
Histological grade			
1	62 (36.5)	108 (63.5)	21.68 (**<0.0001**)
2	374 (48.6)	396 (51.4)	
3	521 (54.8)	429 (45.2)	
Tumour type			
Ductal	866 (51.0)	833 (49)	19.39 (**0.001**)
Lobular^a^	62 (42.2)	85 (57.8)	
Medullary like	26 (81.2)	6 (18.8)	
Special type^a^	19 (38.8)	30 (61.2)	
Miscellaneous	12 (57.1)	9 (42.9)	
NPI			
Good prognostic group	313 (46.0)	367 (54.0)	7.95 (**0.019**)
Moderate prognostic group	580 (52.8)	519 (47.2)	
Poor prognostic group	104 (52.3)	95 (47.7)	
PAM50 subtype			
Luminal A	297 (41.4)	421 (58.6)	165.80 (**<0.00001**)
Luminal B	179 (36.7)	309 (63.3)	
Basal	242 (73.8)	86 (26.2)	
Her2	164 (68.3)	76 (31.7)	
Normal like	112 (56.6)	86 (43.4)	
IntClustMemb			
IntClustMemb 1	62 (44.6)	77 (55.4)	120.96 (**<0.00001**)
IntClustMemb 2	32 (44.4)	40 (55.6)	
IntClustMemb 3	107 (36.9)	183 (63.1)	
IntClustMemb 4	183 (53.4)	160 (46.6)	
IntClustMemb 5	117 (61.6)	73 (38.4)	
IntClustMemb 6	39 (45.9)	46 (54.1)	
IntClustMemb 7	106 (55.8)	84 (44.2)	
IntClustMemb 8	105 (35.1)	194 (64.9)	
IntClustMemb 9	77 (53.1)	68 (46.9)	
IntClustMemb 10	169 (75.1)	56 (24.9)	
ER			
Negative	312 (71.2)	126 (28.8)	97.43 (**<0.00001**)
Positive	665 (44.4)	832 (55.6)	
PR			
Negative	566 (60.3)	373 (39.7)	69.69 (**<0.00001**)
Positive	431 (41.5)	608 (58.5)	
HER2			
Negative	844 (48.8)	887 (51.2)	15.03 (**0.0001**)
Positive	153 (61.9)	94 (38.1)	

Significant *p*-values are highlighted in bold

### MED7 expression and BC biomarkers

For IHC analysis on the Nottingham BC series, the specificity of the antibody was validated with a single specific band at the predicted size (32 kDa; Fig. 1c). MED7 IHC showed nuclear staining with no cytoplasmic or stromal staining (Fig. 1d–f). The *H*-Scores of MED7 nuclear expression did not follow a normal distribution and hence the cut-off point for the MED7 *H*-score for low/high was set by the SPSS programme at the median (*H* score > 130). All cut-offs were set before analysis. Of the 1280 informative cores, 637 (49.8%) had high MED7 expression and 643 (50.2%) showed low expression. Similar to the mRNA observation, protein expression was also associated with good prognostic parameters. High nuclear MED7 expression was associated with smaller tumour size (*p* < 0.0001), lower grade (*p* < 0.0001), lower mitotic scores (*p* < 0.0001), higher tubule formation (*p* = 0.0004) and less nuclear pleomorphism (*p* < 0.0001). Lobular carcinomas showed significantly higher expression of MED7 (*p* = 0.001) in comparison to ductal no special type and medullary subtypes (Table 2). Loss of MED7 protein was correlated with positive LVI status (*p* = 0.04). Overall, a relative lack of MED7 correlated with a poorer NPI (*p* < 0.0001).

**Table 2:** Relationship between nuclear MED7 protein (IHC) and clinicopathological parameters of the Nottingham BC series

Clinicopathological Criteria	MED7 nuclear staining		χ^2^ (*p*-Value)
	Low (%)	High (%)	
Age at diagnosis			
≤50	220 (48.6)	233 (51.4)	0.781 (0.381)
>50	423 (51.1)	404 (48.9)	
Tumour size (cm)			
≤2.0	274 (44.1)	348 (55.9)	19.09 (**<0.0001**)
>2.0	366 (56.3)	284 (43.7)	
Histological grade			
1	82 (39.8)	124 (60.2)	49.341 (**<0.0001**)
2	174 (40.8)	252 (59.2)	
3	383 (60.2)	253 (39.8)	
Tubule formation			
1	26 (39.4)	40 (60.6)	15.589 (**0.0004**)
2	185 (43.8)	237 (56.2)	
3	406 (54.6)	338 (45.4)	
Nuclear pleomorphism			
1	8 (26.7)	22 (73.3)	29.894 (**<0.0001**)
2	195 (41.9)	270 (58.1)	
3	413 (56.2)	322 (43.8)	
Mitotic score			
1	164 (39.8)	248 (60.2)	43.005 (**<0.0001**)
2	110 (44.4)	138 (55.6)	
3	343 (60.0)	229 (40.0)	
Tumour type			
Ductal	551 (51.6)	516 (48.4)	20.575 (**0.001**)
Lobular^a^	41 (35.3)	75 (64.7)	
Medullary like	22 (75.9)	7 (24.1)	
Special type	22 (42.3)	30 (57.7)	
Lymph node stage^b^			
I	398 (51.1)	381 (48.9)	1.853 (0.603)
II	191 (49.6)	194 (50.4)	
III	50 (48.1)	54 (51.9)	
NPI			
Good prognostic group	157 (40.6)	230 (59.4)	23.546 (**<0.0001**)
Moderate prognostic group	358 (53.1)	316 (46.9)	
Poor prognostic group	125 (59.2)	86 (40.8)	
IHC-validated LVI			
Negative	319 (47.5)	352 (52.5)	4.10 (**0.04**)
Positive	196 (54.1)	166 (45.9)	

Significant *p*-values are highlighted in bold.

^a^Tumour type *p*-value reflects association between MED7 and lobular tumours

^b^Lymph node stages 1, 2 and 3 refer to the lymph node staging score incorporated in the Nottingham Prognostic Index, routinely used for breast cancer prognostication: 1 refers to no lymph nodes being involved; 2 refers to 1–3 lymph nodes positive, and 3 >3 lymph nodes positive

On IHC, high nuclear MED7 expression showed significant positive association with ER/PR status (*p* < 0.0001), while negative association was observed with basal cytokeratins CK5/6 (*p* < 0.001) and CK17 (*p* = 0.003). Basal-like BC is highly heterogeneous associated with high grade, poor patient outcome and CK5/6 and CK17 expression. MED7 expression was correlated with other characterised biomarkers on the series, some explored for their known association with ER+luminal subtypes, viz., coactivator-associated arginine methyltransferase 1 (CARM1), an ERα coactivator,^31^ the ER–chromatin interaction regulator Forkhead box protein A1 (FOXA1)^32^ and RAS-like oestrogen regulated growth inhibitor (RERG).^33^ High expression of MED7 was positively associated with these luminal subtype-related bio-markers: CARM1 (*p* = 0.015), RERG (*p* = 0.015), and FOXA1 (*p* < 0.00001). Positive correlations were observed with cell cycle regulatory proteins such as GATA3 (*p* < 0.0001), and STAT3 (*p* < 0.0001), markers also known to be highly expressed in ER+ BC associated with favourable outcome.^34, 35^ Expression of histone modifiers that influence hormone-responsive gene expression in BC^36^ were also positively associated with MED7 expression: viz., histone methylation modifiers at lysine (H3K4Me2: *p* = 0.041; H4K12ac: *p* = 0.004) and arginine residues (H4R3Me2; *p* = 0.048) (Table 3). In contrast, negative correlations were observed with proliferation markers such as Ki67 (*p* = 0.002), epithelial–mesenchymal transition (EMT) markers such as N-cadherin (*p* < 0.0001) and signalling pathway biomarkers like phosphatidylinositol-4,5-bisphosphate 3-kinase catalytic subunit alpha (PIK3CA; *p* = 0.001) and the epidermal growth factor receptor (EGFR; *p* = 0.009).

**Table 3:** Associations of nuclear MED7 IHC expression and other tissue biomarkers within the Nottingham BC series

BC biomarker	MED7 nuclear staining		χ^2^ (*p*-value)
	Low (%)	High (%)	
ER			
Negative	208 (62.8)	123 (37.2)	28.120 (**<0.0001**)
Positive	429 (45.9)	506 (54.1)	
PgR			
Negative	305 (58.7)	215 (41.3)	22.014 (**<0.0001**)
Positive	316 (45.1)	385 (54.9)	
HER2			
Negative	517 (49.3)	532 (50.7)	3.22 (0.085)
Positive	98 (56.6)	75 (43.4)	
CK5/6			
Negative	385 (50.4)	379 (49.6)	12.895 (**<0.001**)
Positive	99 (66.4)	50 (33.6)	
CK17			
Negative	376 (51.8)	350 (48.2)	9.012 (**0.003**)
Positive	72 (67.3)	35 (32.7)	
STAT3			
Negative	356 (57.7)	261 (42.3)	44.140 (**<0.0001**)
Positive	77 (32.4)	161 (67.6)	
GATA3			
Negative	343 (62.4)	207 (37.6)	41.045 (**<0.0001**)
Positive	59 (34.5)	112 (65.5)	
EGFR			
Negative	468 (48.4)	498 (51.6)	7.052 (**0.009**)
Positive	149 (57.8)	109 (42.2)	
PIK3CA			
Negative/low	99 (43.2)	130 (56.8)	13.287 (**0.001**)
Medium	115 (47.7)	126 (52.3)	
High	270 (57.0)	204 (43.0)	
N-cadherin			
Negative	106 (37.7)	175 (62.3)	31.728 (**<0.0001**)
Positive	355 (58.0)	257 (42.0)	
Ki67			
Low	179 (44.8)	221 (55.2)	10.305 (**0.002**)
High	328 (55.1)	267 (44.9)	
H3K4Me2			
Negative	82 (54.3)	69 (45.7)	4.591 (**0.041**)
Positive	115 (43.4)	150 (56.6)	
H4K12ac			
Negative	89 (56.7)	68 (43.3)	8.654 (**0.004**)
Positive	107 (41.8)	149 (58.2)	
H4R3Me2			
Negative	113 (55.1)	92 (44.9)	4.233 (**0.048**)
Positive	115 (45.5)	138 (54.5)	
RERG			
Negative	326 (53.4)	284 (46.6)	5.893 (**0.015**)
Positive	89 (43.6)	115 (56.4)	
CARM1			
Negative	127 (59.1)	88 (40.9)	6.057 (**0.015**)
Positive	85 (46.7)	97 (53.3)	
FOXA1			
Negative	278 (63.5)	160 (36.5)	41.623 (**<0.0001**)
Positive	154 (40.8)	223 (59.2)	

Significant *p*-values are highlighted in bold

### MED7 and association with patient outcome

**Fig. 2:**
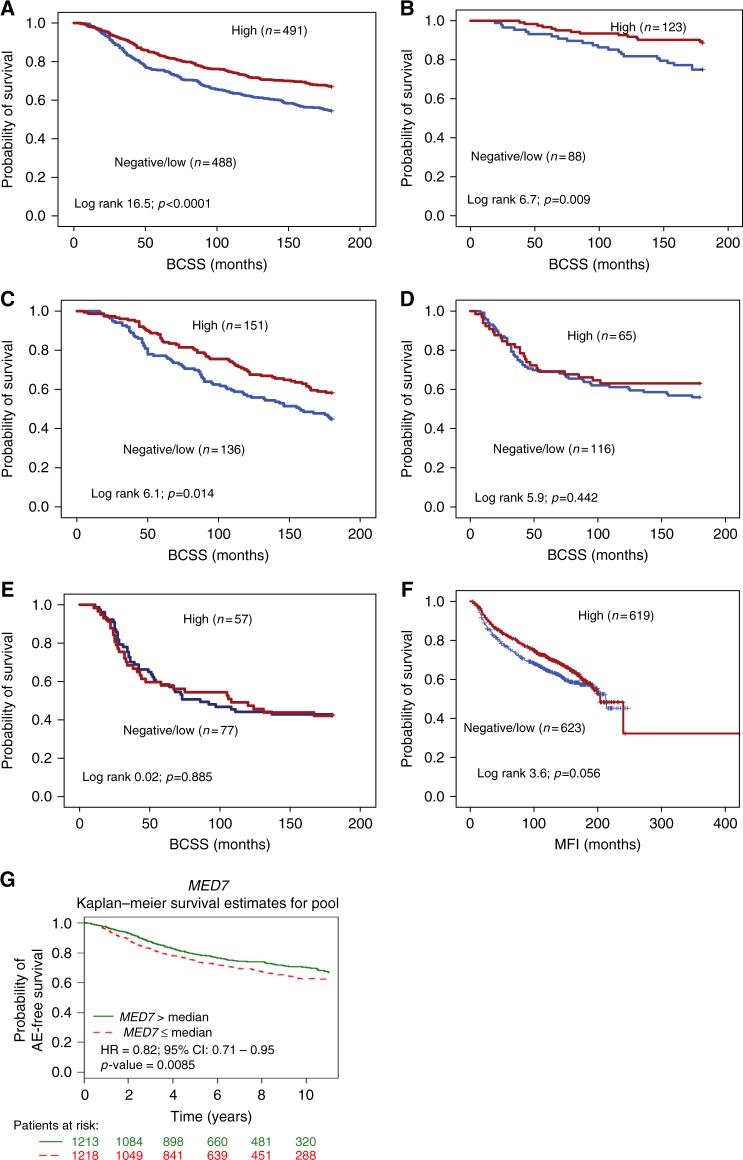
Kaplan–Meier survival plots for MED7 nuclear expression: **a** MED7 vs BCSS in all cases; **b** MED7 vs BCSS in Luminal A; **c** MED7 vs BCSS in Luminal B; **d** MED7 vs BCSS in TPN; **e** MED7 vs BCSS in HER2+; **f** MED7 vs MFI in all cases, and **g** targeted prognostic analyses for MED7 via the BC gene miner in ER+ node-negative patients using Breast Cancer Gene-Expression Miner v4.0

High expression of MED7 protein was predictive of longer BCSS in the whole cohort (*p* < 0.0001, hazard ratio (HR) = 0.66; 95% confidence interval (CI): 0.54–0.81; Fig. 2a), Luminal A (*p* = 0.009, HR = 0.42; 95% CI: 0.21–0.82; Fig. 2b) and Luminal B (*p* = 0.014, HR = 0.66; 95% CI: 0.47–0.92: Fig. 2c) subtypes. There was no association between MED7 protein and outcome in TPN (*p* = 0.442, HR = 0.83; 95% CI: 0.51–1.3: Fig. 2d) and Her2+ subgroups (*p* = 0.885, HR = 1.0; 95% CI: 0.66–1.62: Fig. 2e). However, MED7 expression was not significantly associated with MFI (*p* = 0.056, HR = 0.84; 95% CI: 0.70–1.0: Fig. 2f). In multivariate Cox regression analysis, MED7 protein was a predictor of better BCSS in the whole cohort and Luminal A subtypes. (*p* < 0.01, Table 4).

**Table 4:** Univariate and multivariate analysis: effects of nuclear MED7 expression, LN stage, grade, and LVI

Variable	Univariate			Multivariate		
	HR	95% CI	*p*-Value	HR	95% CI	*p*-Value
Whole cohort						
Stage	2.1	1.9–2.4	**<0.001**	1.8	1.5–2.1	**<0.001**
Grade	2.3	2.0–2.6	**<0.001**	1.9	1.6–2.3	**<0.001**
LVI	2.1	1.8–2.6	**<0.001**	1.5	1.2–1.9	**0.001**
MED7	0.7	0.5–0.8	**<0.001**	0.7	0.6–0.9	**0.007**
Luminal A						
Stage	2.3	1.6–3.4	**<0.0001**	1.9	1.1–3.3	**0.025**
Grade	2.4	1.6–3.6	**<0.0001**	1.4	0.8–2.5	0.204
LVI	1.0	0.5–2.0	0.937	0.8	0.4–1.9	0.663
MED7	0.4	0.2–0.8	**0.012**	0.5	0.2–0.9	**0.028**
Luminal B						
Stage	1.8	1.5–2.2	**<0.001**	1.8	1.3–2.5	**<0.001**
Grade	1.8	1.4–2.3	**<0.001**	2.9	0.9–8.7	0.015
LVI	2.0	1.5–2.7	**<0.001**	1.5	0.9–2.5	0.062
MED7	0.7	0.4–0.9	**0.015**	0.9	0.6–1.4	0.074
TPN						
Stage	1.7	1.3-–2.2	**<0.001**	1.6	1.1–2.3	**0.022**
Grade	1.8	1.4–2.3	0.964	1.9	0.8–2.0	0.812
LVI	2.2	1.4–3.3	**<0.001**	1.7	0.9–3.0	0.085
MED7	0.8	0.5–1.3	0.444	0.9	0.5–1.5	0.673
HER2						
Stage	2.4	1.8–3.1	**<0.001**	1.8	1.2–2.7	**0.002**
Grade	2.9	0.9–8.7	0.057	1.5	0.9–2.7	0.141
LVI	2.1	1.3–3.3	**0.003**	2.4	1.3–4.3	**0.003**
MED7	1.0	0.6–1.6	0.885	1.0	0.6–1.6	0.868

Significant *p*-values are highlighted in bold

### The prognostic impact of MED7 mRNA expression using bc-GenExMiner v 4.0 (Breast Cancer Gene-Expression Miner v 4.0)

Targeted prognostic analyses for *MED7* in LN negative BCs (n = 34 data sets, 2431 patients) indicated that high levels of gene expression correlated with adverse event-free survival (*p* = 0.0085; HR = 0.82; 95% CI: 0.71–0.95, Fig. 2g). As LVI status was not available for these cohorts, adverse event-free survival served as a surrogate for the early metastatic phenotype. Expression analysis for *MED7* with respect to hormone status (ER, PgR and HER2; *n* = 5461; Supplementary Figure 2A-C) corroborates with our analyses on the METABRIC/Nottingham cohorts that higher MED7 correlated with ER/PR-positive status (*p* < 0.0001) and but not with HER2 status (*p* > 0.05). Similarly, interrogation of the BC gene miner data revealed correlations with lower grade (*n* = 3421; *p* < 0.0001) and better NPI (*n* = 1684; *p* = 0.0021).

## Discussion

Despite the plethora of biomarkers studied in BC, the molecular signature underlying LVI in BC is undefined. Differential gene expression analysis in strictly defined LVI strata (morphology supplemented by IHC) in well-validated BC cohorts would potentially provide the ideal opportunity to interrogate key molecules involved in generating the early metastatic phenotype. Using this approach utilising the METABRIC data set, the Mediator subunit MED7 was identified to be negatively related to LVI. To date, there has been relatively little research into MED7 compared with other Mediator subunits with respect to its potential role in BC. Of the other Mediator subunits, MED1 is required for ER-mediated gene transcription and BC cell growth^37^ and has also been implicated in vascular invasion.^13^ On the other hand, recurrent MED12 somatic mutations have been seen in fibroadenomas and phyllodes tumours.^38^ In contrast, the functional relationships of MED7 are little known. The aim of the study was to assess the potential associations between MED7 protein and various clinicopathological variables in primary BC including hormonal receptor status and LVI to investigate its potential as a prognostic tool.

High expression of both MED7 mRNA and protein were significantly associated with better behaving tumour characteristics, viz., low histological grade, older age, good NPI, ER+/PR+ tumours and histological subtypes of good prognosis. This is comparable to studies in other tumours where MED7 downregulation was significantly associated with increased risk of gastrointestinal stromal tumours.^11^ MED7 was preferentially positive in the lobular carcinomas in contrast to ductal or medullary-like tumours. Invasive lobular carcinomas are classically of lower grade with a low rate of mitosis and relatively uniform nuclei.^39, 40^ Many classical-type invasive lobular carcinoma tumours express ER and PR,^41^ both of which were significantly positively associated with MED7 expression. It was hypothesised that low-grade BCs develop through a different pathway from high-grade tumours that may involve alterations in the expression of ER and altered genetic profiles in low-grade compared with high-grade tumours.^42^ This could implicate MED7 as having a putative role in low-grade ER+ tumourigenic pathways. Moreover, high *MED7* mRNA expression level was significantly associated with the ER-positive integrative clusters 3 and 8, which had the most favourable clinical outcome in the METABRIC study.^5^

There are several pathways by which ER is able to activate gene transcription. In the ER-mediated pathway, dimerised ER directly binds to DNA sequences called oestrogen response elements in relevant activated genes. However, ER is also known to use non-classical pathways to activate these genes either via activator protein 1 or via specificity protein 1 (Sp-1). MED7 acts as a co-regulator for Sp1 activity^43^, and it is therefore possible that MED7 acts within the non-classic Sp-1 pathway of ER gene activation. The biomarkers characterised on the Nottingham Primary series also serve as indicators of possible molecular networks in ER+ tumours where MED7 may be an interacting partner. Markers known to be overexpressed in luminal BC, viz., CARM1,^31^ RERG^44^ and FOXA1^45^, revealed significant positive association with MED7 as also luminal CKs, steroid receptors and cell cycle inhibitors (p21 and p27), which are associated with good prognostic characteristics. MED7’s positive association with luminal markers indicate its role in better behaving tumours. However, the role of MED7 within the ER-related pathways may be quite complex, depending on the specific interacting partner. For example, in this study, MED7 expression was found to be negatively associated with EGFR expression. On one hand, it is known that EGFR overexpression in BC is associated with increased tumour size and worse patient outcomes and negatively correlates with ER status,^46^ explaining the observed negative association with MED7. However, it is also known that activation of EGFR by EGF triggers phosphorylation of mitogen-activated protein kinase and extracellular signal–regulated kinase, which in turn causes phosphorylation of Ser118 of the AF-1 domain of ERα, resulting in ER transactivation.^47^ This transactivation can also occur via the PIK3CA and AKT pathway. Given the inverse relationship between MED7 and EGFR, MED7 may reduce EGFR-mediated ligand-independent ER activation. Its inverse relationship to PIK3CA is also another interesting link to investigate further within the milieu of intersecting ER-regulating pathways as other studies indicate that PIK3CA mutations are strongly associated with ER-positive tumours with better prognostic characteristics.^48^ ER+ BCs undergo extensive chromatin remodelling and histone modifications for hormone-responsive gene expression. For instance, overexpression of H4K12ac was associated with ER+ cells, and these levels were further increased by oestrogen treatment.^36^

High levels of *MED7* mRNA or protein was associated with a better prognosis in BC. On both univariate and multivariate analysis, MED7 expression was significantly associated with an improved long-term prognosis. In terms of the poor prognostic indicator, LVI,^49^ MED7 was negatively related with LVI, implying its protective role in BC. Given the strong association between MED7 and ER-positive low-grade luminal BCs, it is more likely that this correlation is a passenger effect rather than a driver event. Also, the overall correlation with good prognosis in the whole cohort seems to stem chiefly from MED7’s strong prognostic correlations in ER+Luminal A tumours. Though the prognostic effect of MED7 is not observed in ER− tumours, the prognostic value in ER-positive BCs is potentially useful. Some ER-positive tumours are known to recur in the long run; as MED7 is of prognostic significance over a long time span, this may help discriminate between good vs poorly performing ER-positive tumours. In this study, the negative correlations with N-cadherin, CK5/6 and CK17 indicates that MED7 expression is not associated with aggressive BCs. N-cadherin gain is an EMT-associated phenomenon contributing to BC aggressiveness^50^ and tumour invasion and MED7 may be protective against BC cells acquiring an EMT-prone phenotype. CKs were strongly associated with high histological grade (III), ER− and PgR− status and worse patient outcome^51^ and their negative association with MED7 further strengthens its role in non-basal-type BC.

There is an increasing focus towards the role of molecular approaches in the classification of BC as well as the use of epigenetics to devise new prognostic markers and predictive tools.^52^ Recent research has indicated that epigenetic alterations such as DNA methylation and histone modifications play a role in the development of various cancers including BC, as these changes can affect multiple gene networks and are able to influence many cellular processes related to tumourigenesis.^53^ Histone modification methods, acetylation^54^ and methylation^55^ in particular are known to impact on gene expression in cancer including BC.^56^ MED7 was significantly positively associated with modified histone marks H3K4Me2 and H4K12ac (methylation of lysine) and H4R3Me2 (methylation of arginine). High H4R3Me2, with which MED7 was significantly associated, was associated with good prognosis and a longer disease-free survival and with luminal-type tumours and hormone receptor expression.^56^ Positive vascular invasion, associated with lower MED7 levels, is also known to be associated with low levels of other histone marks like H4K16ac^56^. It may be conjectured that MED7 may be involved in some part of the histone modification process or is recruited to genes that have been modified by histones to confer a better overall prognosis.

This study revealed and confirmed that MED7 was associated with good prognostic characteristics and better long-term survival outcome in BC. Morphologically, it is significantly associated with invasive lobular cancers. Overexpression of MED7 particularly appears to play a significant role in ER+luminal subtype of BC, and given its association with multiple ER-related markers, further functional assessment is necessary to reveal the specific role played by this Mediator protein in these ER-positive tumours. The current study suggests a multi-functional role of MED7 in invasive BC biology and validates the utility of multi-platform approaches (global expression profiling, complemented by immunohistochemistry) in prognostic biomarker discovery.

## Electronic supplementary material

Supplementary Table 1

Supplementary Table 2

Supplementary Table 3

Supplementary Table 4

Supplementary Figure 1

Supplementary Figure 2
