# Supplementary material for: Mediator complex (MED) 7: a biomarker associated with good prognosis in invasive breast cancer, especially ER+ luminal subtypes
Source: Br J Cancer. 2018 Mar 28;118(8):1142–51. doi: 10.1038/s41416-018-0041-x (PMC5931067; doi:10.1038/s41416-018-0041-x)
Supplement: Supplementary file 1 — Supplementary Table 1 [file 41416_2018_41_MOESM1_ESM.docx]

Supplementary Table1: Summary of clinico-pathological characteristics for the METABRIC cohort.

| **Clinicopathological Parameter** | **Frequency (%)** | **Clinicopathological Parameter** | **Frequency (%)** |
| --- | --- | --- | --- |
| **Age at Diagnosis** | | **Tumour Size** | |
| ≤50 years | 424 (21.9) | ≤2.0cm | 858 (43.8) |
| >50 years | 1511(78.1) | >2.0cm | 1102 (56.2) |
| **Menopausal Status** | | **Grade** | |
| Pre | 436 (22.0) | 1 | 170 (9.0) |
| Post | 1533 (78.0) | 2 | 770 (40.7) |
|  |  | 3 | 952 (50.3) |
| **Stage** | | **NPI** | |
| 1 | 1035 (49.1) | Good Prognostic Group | 680 (34.3) |
| 2 | 622 (34.9) | Moderate Prognostic Group | 1101 (55.6) |
| 3 | 316 (16.0) | Poor Prognostic Group | 199 (10.1) |
| **Tumour Type** | | **Tumour Type** | |
| Ductal | 1643 (83.8) | Special-type | 113 (5.8) |
| Lobular | 147 (7.5) | Miscellaneous | 25 (1.3) |
| Medullary-like | 32 (1.6) |  |  |
| **ER status** | | **HER2 Status** | |
| Positive | 1506 (76.1) | Positive | 247 (12.5) |
| Negative | 474 (23.9) | Negative | 1733 (87.5) |
| **PgR Status** | | **Triple Negative Status** | |
| Positive | 1040 (52.5) | Triple Negative | 276 (16.1) |
| Negative | 940 (47.5) | Non-Triple Negative | 1661 (83.9) |
