# Supplementary material for: Mediator complex (MED) 7: a biomarker associated with good prognosis in invasive breast cancer, especially ER+ luminal subtypes
Source: Br J Cancer. 2018 Mar 28;118(8):1142–51. doi: 10.1038/s41416-018-0041-x (PMC5931067; doi:10.1038/s41416-018-0041-x)
Supplement: Supplementary file 2 — Supplementary Table 2 [file 41416_2018_41_MOESM2_ESM.docx]

Supplementary Table2: Datasets (Study code and reference) included in the online analysis for MED7 on the BC-GenExMiner v4.0 (Breast Cancer Gene-Expression Miner v4.0 online dataset).

| **Study code** | **Reference** | |
| --- | --- | --- |
| **Rosetta2002** | [Van de Vijver *et al.*, 2002](http://www.ncbi.nlm.nih.gov/pubmed/12490681) | |
| **GSE1378** | [Ma *et al.*, 2004](http://www.ncbi.nlm.nih.gov/pubmed/15193263) |  |
| **GSE2603** | [Minn *et al.*, 2005](http://www.ncbi.nlm.nih.gov/pubmed/16049480) | |
| **GSE1456** | [Pawitan *et al.*, 2005](http://www.ncbi.nlm.nih.gov/pubmed/16280042) | |
| **GSE2034** | [Wang *et al.*, 2005](http://www.ncbi.nlm.nih.gov/pubmed/15721472) | |
| **GSE2741** | [Weigelt *et al.*, 2005](http://www.ncbi.nlm.nih.gov/pubmed/16230372) | |
| **GSE3143** | [Bild *et al.*, 2006](http://www.ncbi.nlm.nih.gov/pubmed/16273092) | |
| **E_TABM_158** | [Chin *et al.*, 2006](http://www.ncbi.nlm.nih.gov/pubmed/17157792) | |
| **GSE4922** | [Ivshina *et al.*, 2006](http://www.ncbi.nlm.nih.gov/pubmed/17079448) | |
| **GSE8757** | [Chin *et al.*, 2007](http://www.ncbi.nlm.nih.gov/pubmed/17925008) | |
| **GSE7390** | [Desmedt *et al.*, 2007](http://www.ncbi.nlm.nih.gov/pubmed/17545524) | |
| **GSE6532** | [Loi *et al.*, 2007](http://www.ncbi.nlm.nih.gov/pubmed/17401012) | |
| **GSE5327** | [Minn *et al.*, 2007](http://www.ncbi.nlm.nih.gov/pubmed/17420468) | |
| **E_UCON_1** | [Naderi *et al.*, 2007](http://www.ncbi.nlm.nih.gov/pubmed/16936776) | |
| **GSE7378** | [Zhou *et al.*, 2007](http://www.ncbi.nlm.nih.gov/pubmed/17407600) | |
| **GSE7849** | [Anders *et al.*, 2008](http://www.ncbi.nlm.nih.gov/pubmed/18167534) | |
| **GSE9893** | [Chanrion *et al.*, 2008](http://www.ncbi.nlm.nih.gov/pubmed/18347175) | |
| **GSE9195** | [Loi *et al.*, 2008](http://www.ncbi.nlm.nih.gov/pubmed/18498629) | |
| **GSE11121** | [Schmidt *et al.*, 2008](http://www.ncbi.nlm.nih.gov/pubmed/18593943) | |
| **GSE10510** | [Calabrò *et al.*, 2009](http://www.ncbi.nlm.nih.gov/pubmed/18592372) | |
| **GSE16391** | [Desmedt *et al.*, 2009](http://www.ncbi.nlm.nih.gov/pubmed/19573224) | |
| **GSE12093** | [Zhang *et al.*, 2009](http://www.ncbi.nlm.nih.gov/pubmed/18821012) | |
| **GSE22133** | [Jönsson *et al.*, 2010](http://www.ncbi.nlm.nih.gov/pubmed/20576095) | |
| **GSE19615** | [Li *et al.*, 2010](http://www.ncbi.nlm.nih.gov/pubmed/20098429) | |
| **GSE17907** | [Sircoulomb *et al.*, 2010](http://www.ncbi.nlm.nih.gov/pubmed/20932292) | |
| **GSE22219** | [Buffa *et al.*, 2011](http://www.ncbi.nlm.nih.gov/pubmed/21737487) | |
| **GSE20711** | [Dedeurwaerder *et al.*, 2011](http://www.ncbi.nlm.nih.gov/pubmed/21910250) | |
| **GSE26971** | [Filipits *et al.*, 2011](http://www.ncbi.nlm.nih.gov/pubmed/21807638) | |
| **GSE25055** | [Hatzis *et al.*, 2011](http://www.ncbi.nlm.nih.gov/pubmed/21558518) | |
| **GSE20685** | [Kao *et al.*, 2011](http://www.ncbi.nlm.nih.gov/pubmed/21501481) | |
| **GSE21653** | [Sabatier *et al.*, 2011](http://www.ncbi.nlm.nih.gov/pubmed/20490655) | |
| **GSE16987** | [Wang *et al.*, 2011](http://www.ncbi.nlm.nih.gov/pubmed/21939527) | |
| **GSE33926** | [Kuo *et al.*, 2012](http://www.ncbi.nlm.nih.gov/pubmed/23049873) | |
| **GSE45255** | [Nagalla *et al.*, 2013](http://www.ncbi.nlm.nih.gov/pubmed/23618380) | |
