# Supplementary material for: Mediator complex (MED) 7: a biomarker associated with good prognosis in invasive breast cancer, especially ER+ luminal subtypes
Source: Br J Cancer. 2018 Mar 28;118(8):1142–51. doi: 10.1038/s41416-018-0041-x (PMC5931067; doi:10.1038/s41416-018-0041-x)
Supplement: Supplementary file 4 — Supplementary Table 4 [file 41416_2018_41_MOESM4_ESM.docx]

Supplementary Table 4: Biomarkers characterised in the Nottingham Tenovus Primary Series with antibodies used, source, dilutions and cut-offs for categorisation.

| **Antibody [clone]** | **Supplier** | **Dilution** | **Categorical cut points** |
| --- | --- | --- | --- |
| ER [1D5] | DAKO | 1:150 | Negative (0),  Positive (>1) |
| PgR[PgR 636] | DAKO | 1:100 | Negative (0),  Positive (>1) |
| HER2 [cerbB-2] | DAKO | 1:250 | Negative (<10),  Positive (≥10) |
| Ck5/6 [ D5/16134] | Boehringer Biochemica | 1:100 | Negative (<10),  Positive (≥10) |
| Ck17[E3] | Abcam | 1:20 | Negative (<10),  Positive (≥10) |
| Trans-acting T-cell-specific transcription factor (GATA3) [HG3-31] | Santa Cruz Biotechnology | 1:80 | Negative/Low (<60), Positive (≥60) |
| Co-activator associated arginine methyl transferase (CARM1) [clone NB100 -1817] | NOVUS | 1:300 | Negative/Low (<30),  Moderate (30-149),  High (150-300) |
| EGFR[31G7] | lone 31G7, Invitrogen | 1/30 | ≥10% |
| STAT3 | Abcam | 1:150 | 0-30 Negative/Low  and >30 High |
| Phosphatidylinositol-3 kinase [HPA009985] | Sigma | 1:50 | Negative (<30),  Low (30-100),  High (101-300) |
| Ki-67[MIB1] | Sigma | 1:100 | Negative (<14),  Positive (>14) |
| N-Cadherin [C3865] | Abcam | 4 ug/ml | Negative/Low (<100),  High (>100) |
| Anti-Histone H4 acetyl K12 [H4K12ac] | Abcam | 1:800 | Negative/Low (<100),  High (>100) |
| Anti-Histone H3 (di methyl K4 [H3K4Me2; [C3865] | Abcam | 1:200 | Negative/Low (<100),  High (>100) |
| Anti-Histone H4 (di methyl R3) antibodyH4R3Me2 | Abcam | 1:200 | Negative/Low (<100),  High (>100) |
| RERG (Ras-like, oestrogen-regulated, growth-inhibitor | Proteintech | 1:20 | Negative (0),  Positive (1) |
| Forkhead box protein A1 (FOXA1) [clone 2F83 | ABCAM | 1:2000 | Negative (<10),  Positive (≥10) |
