# Supplementary material for: Mediator complex (MED) 7: a biomarker associated with good prognosis in invasive breast cancer, especially ER+ luminal subtypes
Source: Br J Cancer. 2018 Mar 28;118(8):1142–51. doi: 10.1038/s41416-018-0041-x (PMC5931067; doi:10.1038/s41416-018-0041-x)
Supplement: Supplementary file 5 — Supplementary Figure 1 [file 41416_2018_41_MOESM5_ESM.pptx]

## Slide 1
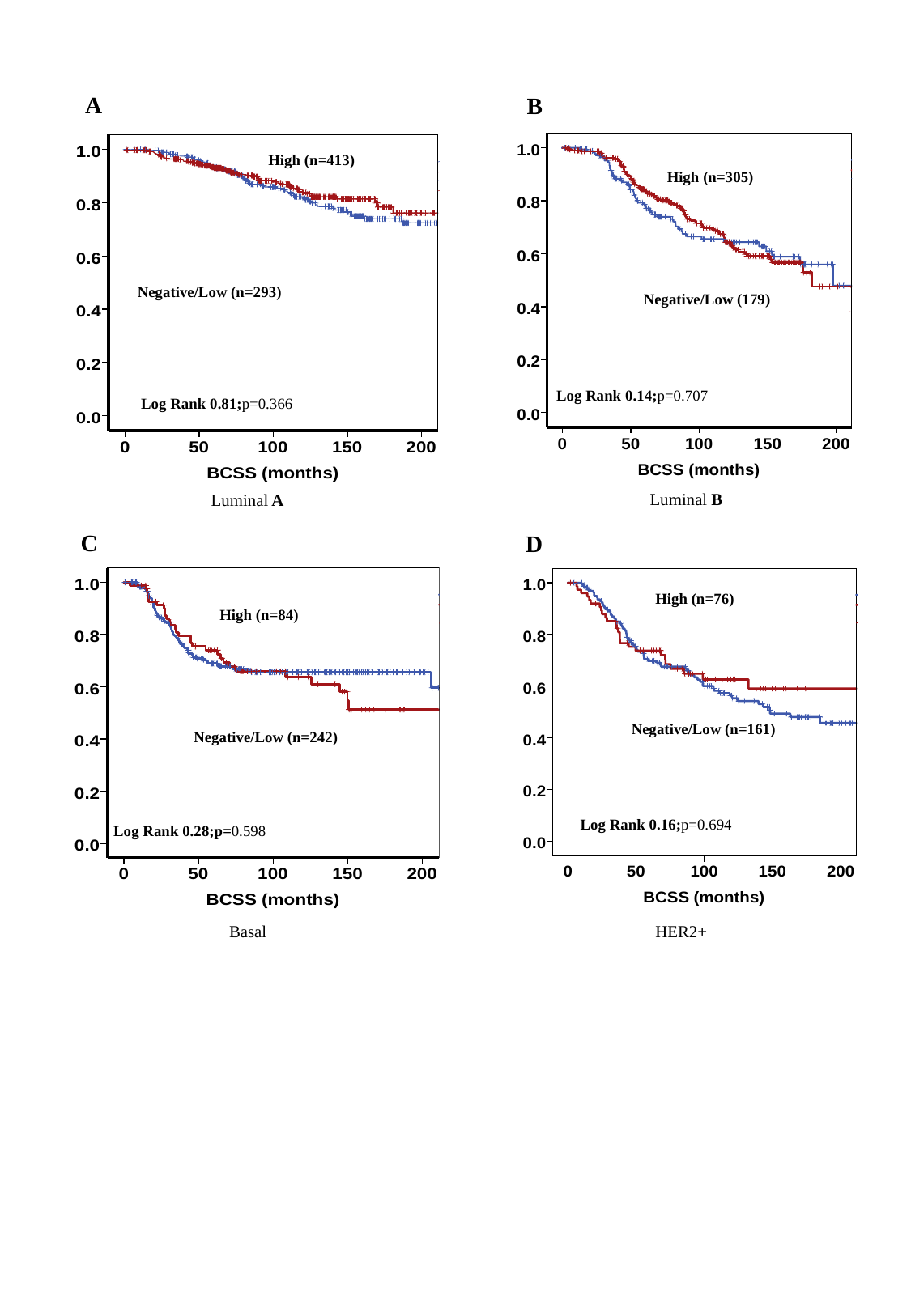

A
High (n=413)
Negative/Low (n=293)
Log Rank 0.81;p=0.366
Luminal A
B
High (n=305)
Negative/Low (179)
Log Rank 0.14;p=0.707
Luminal B
D
High (n=76)
Negative/Low (n=161)
Log Rank 0.16;p=0.694
HER2+
C
High (n=84)
Negative/Low (n=242)
Log Rank 0.28;p=0.598
Basal
