# Supplementary figures and images for: Mediator complex (MED) 7: a biomarker associated with good prognosis in invasive breast cancer, especially ER+ luminal subtypes
Source: Br J Cancer. 2018 Mar 28;118(8):1142–51. doi: 10.1038/s41416-018-0041-x (PMC5931067; doi:10.1038/s41416-018-0041-x)

## Slide 1
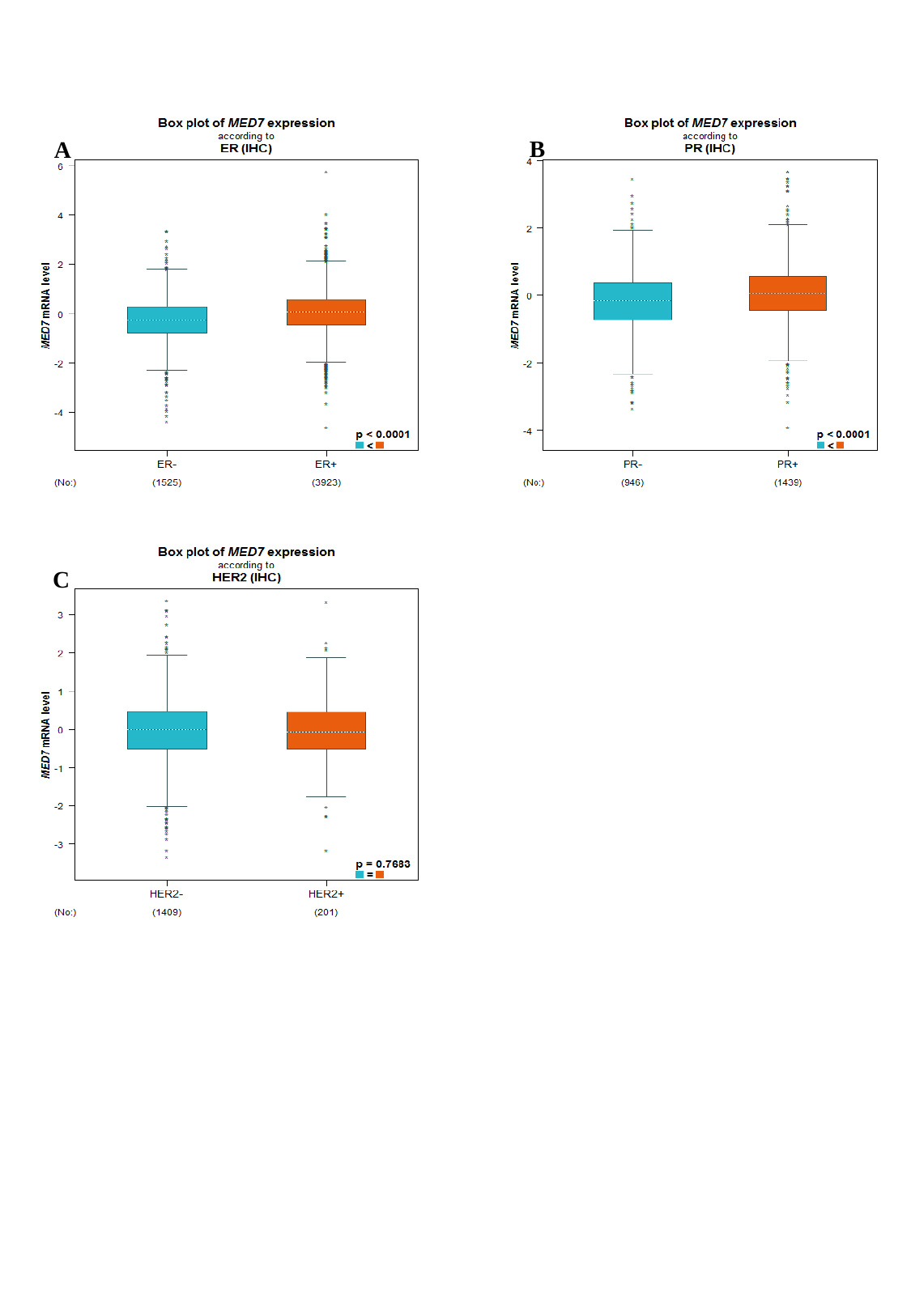

A
B
C

Supplement: Supplementary file 6 — Supplementary Figure 2 [file 41416_2018_41_MOESM6_ESM.pptx]
